# Metagenomics to Identify Viral Communities Associated with Porcine Respiratory Disease Complex in Tibetan Pigs in the Tibetan Plateau, China

**DOI:** 10.3390/pathogens13050404

**Published:** 2024-05-13

**Authors:** Long Zhou, Han Zhou, Yandi Fan, Jinghao Wang, Rui Zhang, Zijing Guo, Yanmin Li, Runmin Kang, Zhidong Zhang, Danjiao Yang, Jie Liu

**Affiliations:** 1 College of Animal & Veterinary Sciences, Southwest Minzu University, Chengdu 610041, China; zhoulongscu@163.com (L.Z.); zh9977zh@163.com (H.Z.); fyd105109@163.com (Y.F.); adlifuwjh@163.com (J.W.); zhangrui@swun.edu.cn (R.Z.); guozijing@swun.edu.cn (Z.G.); liyanmin@swun.edu.cn (Y.L.); jieliuhzau@163.com (J.L.); 2 Key Laboratory of Ministry of Education and Sichuan Province for Qinghai-Tibetan Plateau Animal Genetic Resource Reservation and Utilization, Chengdu 610041, China; 3 Sichuan Animal Science Academy, Sichuan Provincial Key Laboratory of Animal Breeding and Genetics, Chengdu 610066, China; angelina_0708@hotmail.com; 4 Institute of Animal Science of Ganzi Tibetan Autonomous Prefecture of Sichuan Province, Kangding 626000, China

**Keywords:** porcine respiratory disease complex, metagenomics, viral community, Tibetan pig

## Abstract

Tibetan pig is a unique pig breed native to the Qinghai–Tibet Plateau. To investigate viral communities associated with porcine respiratory disease complex (PRDC), 167 respiratory samples were collected from Tibetan pigs in the Ganzi Tibetan autonomous prefecture of Sichuan province. Following library construction and Illunima Novaseq sequencing, 18 distinct viruses belonging to 15 viral taxonomic families were identified in Tibetan pigs with PRDC. Among the 18 detected viruses, 3 viruses were associated with PRDC, including porcine circovirus type 2 (PCV-2), Torque teno sus virus (TTSuV), and porcine cytomegalovirus (PCMV). The genomic sequences of two PCV-2 strains, three TTSuV strains, and one novel Porprismacovirus strain were assembled by SOAPdenovo software (v2). Sequence alignment and phylogenetic analysis showed that both PCV-2 strains belonged to PCV-2d, three TTSuVs were classified to TTSuV2a and TTSuV2b genotypes, and the Porprismacovirus strain PPMV-SCgz-2022 showed a close genetic relationship with a virus of human origin. Recombination analysis indicated that PPMV-SCgz-2022 may have originated from recombination events between Human 16,806 × 66-213 strain and Porcine 17,668 × 82-593 strain. Furthermore, the high proportion of single infection or co-infection of PCV2/TTSuV2 provides insight into PRDC infection in Tibetan pigs. This is the first report of the viral communities in PRDC-affected Tibetan pigs in this region, and the results provides reference for the prevention and control of respiratory diseases in these animals.

## 1. Introduction

Porcine respiratory disease complex (PRDC) is the most common and costly problem in the global swine industry, accounting for 10 to 40% of morbidity and 2 to 20% of mortality in intensive pig farms [1]. PRDC is characterized by retarded growth, increased mortality and costs due to production failure and the associated medicinal/vaccination needs [2]. The primary respiratory viruses are porcine reproductive and respiratory syndrome virus (PRRSV), porcine circovirus type 2 (PCV-2), porcine pseudorabies virus (PRV), swine influenza virus (SIV), and African swine fever virus (ASFV) [3,4,5]. Recent studies have suggested that parvoviruses, bocaviruses and torque teno sus virus (TTSuV) are identified as the novel pathogens associated with PRDC [6,7,8].

Tibetan pig is a unique Chinese indigenous pig breed that lives on the Qinghai–Tibet Plateau at elevations of over 3000 m above sea level [9]. These animals live in this harsh and cold highland environment and usually graze freely. They are typically in close proximity to other livestock such as yaks, Tibetan sheep and goats, providing opportunities for cross-species viral transmission [10]. Tibetan pigs are also kept in close contact with local herdsman, with risk of zoonotic transmission [11,12]. Due to the remote location and extremely harsh environmental conditions of the Tibetan plateau, few studies have examined viral respiratory diseases in Tibetan pigs.

With the recent development of high-throughput sequencing and viral metagenomic analytical methods, efforts have been made to characterize the complex viral communities in clinical samples [13,14]. To date, this approach has been applied to study viral communities in serum, plasma, feces, respiratory secretions, and tissue samples [15,16,17,18]. Deep sequencing, multiplex PCR, and microarray have been used to examine PRDC-related viruses in domestic pigs [6,8,19,20,21]; however, little work has been carried out to characterize these viruses in Tibetan pigs. The pig industry is one of the largest agricultural sectors of the Ganzi Tibetan autonomous prefecture economy, with approximately 100 thousand Tibetan pigs currently being raised. To investigate the respiratory viral communities in Tibetan pigs, 167 respiratory samples including 66 nasal swabs were collected from clinical healthy Tibetan pigs and 101 nasal swabs, lung tissues and alveolar lavage fluids were collected from Tibetan pigs with PRDC. These samples collected from 23 pig farms were used to investigate the detection rates for viruses identified in animals with PRDC to determine the main cause of PRDC by PCR/RT-PCR method.

## 2. Materials and Methods

### 2.1. Ethics Statement

This study was approved by the Institutional Animal Care and Use Committee (IACUC) of the College of Animal & Veterinary Sciences, Southwest Minzu University, China (12 October 2020, Certification No.: SYXK2020-178).

### 2.2. Sample Collection

From 2021 to 2022, 167 respiratory samples were collected from 23 Tibetan pig farms in four counties (Luding, Kangding, Daocheng, and Xiangcheng) from Ganzi Tibetan autonomous prefecture of Sichuan province. Among these samples, 66 nasal swabs were collected from asymptomatic Tibetan pigs from 6 pig farms, and 101 samples were collected from Tibetan pigs (in 17 pig farms) that had been associated with PRDC (i.e., rhinorrhea, cough, shortness of breath, dyspnea, and depression), with nasal swabs (n = 70), lung tissues (n = 23) and alveolar lavage fluids (n = 8) collected. The animals were selected as either PRDC cases or asymptomatic pigs based on the assessment by a local veterinarian. The major pathological lesions observed in the lungs were characterized by diffuse bleeding, edema, hyperemia and interstitial thickening. All collected respiratory samples were added to viral transport medium (VTM, hopebio, Qingdao, China) and stored at −80 °C.

### 2.3. Sample Treatment and Nucleic Acid Extraction

The samples were subjected to a series of pre-treatments before extraction of nucleic acids. Briefly, VTM supernatants from nasal swabs, lung tissues and alveolar lavage fluids were collected. Two pooled samples were assembled using 50 mL of each supernatant from the respiratory samples. The mixed pooled samples were passed through a 200 nm filter (Millipore, Billerica, MA, USA), and the resulting filtrates were concentrated using an Ultra 50K ultrafiltration tube (Millipore, USA). The 2 mL filtrates were incubated at 37 °C for 90 min in a cocktail containing DNase and RNase enzymes (TaKaRa, Dalian, China). Total viral DNA was prepared from the samples using QIAamp Viral DNA Mini Kit (QIAGEN, Hilden, Germany). Total viral RNAs were extracted from three pooled samples using a QIAamp Viral RNA Mini Kit (QIAGEN, Germany) following the manufacturer’s protocol. Reverse transcription was performed using SuperScript III reverse transcriptase (RT) (Invitrogen, Carlsbad, CA, USA) and random hexamers (Invitrogen, USA) following the supplier’s guidelines. Finally, the viral DNA and cDNAs from two pooled samples were sent to TP-Bio Co. Ltd. (Shanghai, China) for library construction and high-throughput Illunima Novaseq sequencing.

### 2.4. Library Construction and High-Throughput Sequencing

The obtained cDNAs from three pooled samples were ultrasonicated to generate fragments ~450 bp in length. Three paired-end (PE) libraries were constructed by DNA fragments end repair and adaptor ligation and sequenced using Illumina’s Novaseq 6000 platform. The Illumina-generated raw data were filtered using Trimmatic software (v0.36) to trim adaptor-related reads, low-quality reads, reads with a high proportion of N-bases (>10%), and short-length reads (<75 bp), resulting in high-quality clean data. The porcine genomic and bacterial reads were removed from the cleaned data using BWA, and the obtained reads were de novo assembled using Megahit software (v1.0). The assembled viral contigs from each pool were aligned with sequences in the nucleic acid and protein databases using BLASTn and BLASTx. The taxonomies of the sequences with the best BLAST values (>90% overlap identity) were selected and used for further grouping and analyses. The viral abundances were calculated using SOAP aligner software (v2) [22]. Reads representing different viruses were individually identified from clinical samples by specific PCR and Sanger sequencing.

### 2.5. Sequence Comparison, Phylogenetic and Recombination Analysis

The open reading frames of the viral nucleotide and amino acid sequences were analyzed using DNAstar software (version 7.0) [23]. Phylogenetic analyses were conducted in MEGAX software (v10) with the Kimura 2-parameter and a nucleotide substitution model, and Maximum-likelihood (ML) trees were constructed from nucleotide sequences using the ClustalW multiple alignment algorithm [24]. The selected viral reference sequences were downloaded from GenBank. The bootstrap values were evaluated from 1000 replicates. In addition, the SimPlot software (v3.5.1) [25] and Recombination Detection Program (v4) [26] were used to detect recombination events and recombination breakpoints within the genomes of viruses. Recombination events were considered significant if confirmed by at least five of seven methods (RDP, Bootscan, GENECONV, MaxChi, Chimaera, SiScan, and 3Seq) with *p* < 0.01. Furthermore, ML trees were generated from each recombinant fragment to confirm the putative recombination events.

### 2.6. Virus Detection Rates in Tibetan Pigs

To detect the viral prevalence in Tibetan pigs with PRDC, total nucleic acid was extracted using a QIAamp Viral DNA Mini Kit (QIAGEN, Germany). Specific primers were designed for each virus based on the Illumina sequencing data. The PCRs using specific primer pairs (Supplementary Table S1) were performed using Best W5 HiPer High-Fidelity DNA Polymerase PCR Red mix (Mei5bio, Beijing, China). DNA (2 μL) from each clinical respiratory sample was used in each reaction. Amplification followed the following cycling profile: a denaturation of 98 °C for 2 min followed by cycles of 98 °C for 8 s, 50–64 °C for 25 s and 72 °C for 15 s. The amplified PCR products were detected on 1.0% agarose gel. PCR products were purified and cloned into pMD19-T vector (TaKaRa, Dalian, China) and sequenced at Sangon Biotech (Shanghai, China).

## 3. Results

### 3.1. Viral Metagenomics

**Figure 1:**
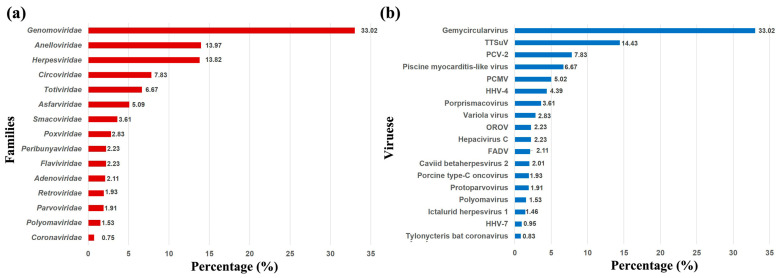
Families (**a**) and species (**b**) classification and percentage of virus sequences detected from respiratory samples from Tibetan pigs.

Using high-throughput sequencing, a total of 110,687,546 reads were obtained from the mixed pooled samples of Tibetan pigs. Approximately 2.21% (2,446,194 reads) of the sequence reads from the clinical respiratory samples mapped to mammalian-associated viral sequences. Metagenomics analysis revealed that the viruses in Tibetan pigs from asymptomatic pigs were mainly bacteriophages, and only a few sequence reads of Mardivirus in *Herpesviridae* (1.54%) were mapped to mammalian-associated viral sequences. Members of fifteen virus families were identified, as follows, in order of sequence read abundance: *Genomoviridae* (33.02% of all reads), *Anelloviridae* (13.97%), *Herpesviridae* (13.82%), *Circoviridae* (7.83%), *Totiviridae* (6.67%), *Asfarviridae* (5.09%), *Smacoviridae* (3.61%), *Poxviridae* (2.83%), *Peribunyaviridae* (2.23%), *Flaviviridae* (2.23%), *Adenoviridae* (2.11%), *Retroviridae* (1.93%), *Parvoviridae* (1.91%), *Polyomaviridae* (1.53%), and *Coronaviridae* (0.75%) (Figure 1a). Eighteen distinct viruses were identified in these fifteen families, in order of sequence read abundance: Gemycircularvirus (33.02% of all reads), Torque teno sus virus (TTSuV, 14.43%), Porcine circovirus type 2 (PCV-2, 7.83%), Piscine myocarditis-like virus (6.67%), Porcine cytomegalovirus (PCMV, 5.02%), Human gammaherpesvirus 4 (HHV-4, 4.39%), Porprismacovirus (3.61%), Variola virus (2.83%), Orthobunyavirus (OROV, 2.23%), Hepacivirus C (2.23%), Fowl aviadenovirus (FADV, 2.11%), Caviid betaherpesvirus 2 (2.01%), Porcine type-C oncovirus (1.93%), Protoparvovirus (1.91%), Polyomavirus (1.53%), Ictalurid herpesvirus 1 (1.46%), Human betaherpesvirus 7 (HHV-7, 0.95%), and Tylonycteris bat coronavirus (0.83%) (Figure 1b). These results show that the viral communities are complex and diverse in Tibetan pigs with PRDC.

### 3.2. Viral Genome Assembly, Sequence Alignment and Phylogenetic Analysis

The contigs from the 18 different identified viruses were de novo assembled using SOAP assembly software (v2), from which six genomic sequences (three strains of TTSuV, two strains of PCV-2, and a Porprismacovirus strain), were assembled using the corresponding viral sequence contigs.

#### 3.2.1. TTSuV

A total of 15,972,212 reads with sequences corresponding to TTSuV were detected in the pooled respiratory samples of Tibetan pigs. Three nearly complete genomes of TTSuV were assembled and designated TTSuV2-1/2022/SCgz/China (2815 bp), TTSuV2-2/2022/SCgz/China (2578 bp) and TTSuV2-3/2022/SCgz/China (2757 bp). These sequences were submitted to the GenBank database (accession no. OQ874787-OQ874789). Similarity analyses revealed that the three TTSuV strains shared 56.2~74.9% nucleotide sequence identity with each other and 55.8~92.5%/55.6~97.9% identity to reference genome sequences of TTSuV 2a/2b strains, but only 34.5% to 39.7% identity to TTSuV 1 reference strains (Table 1). Thus, the three TTSuV strains identified in Tibetan pigs belong to type 2 TTSuV.

**Table 1:** Nucleotide sequence identity values for different regions of three TTSuV strains identified in Tibetan pigs compared with nine TTSuV reference strains.

		GD/China/2009/TTV1/17	TTV1Bj2-1	SH0822/2008	pork_298	lung3	TTV2Ln13	38E19	TTV2Bj1-2	TTV2Jl1
		TTSuV 1			TTSuV 2a			TTSuV 2b		
		Pairwise % Identity to TTSuV (nt)								
Complete genome	TTSuV2-1/2022/SCgz/China	35.8	34.7	34.5	55.9	55.9	55.8	**96.1** ^1^	56.3	55.6
	TTSuV2-2/2022/SCgz/China	39.7	39.1	37.3	75.1	79.1	74.7	55.9	**97.9**	87.7
	TTSuV2-3/2022/SCgz/China	39.2	38.8	38.5	**92.5**	80.3	72.6	55.8	75.1	74.7
ORF1	TTSuV2-1/2022/SCgz/China	34.7	33.4	34.5	57.1	56.2	55.9	**96.5**	57.0	56.0
	TTSuV2-2/2022/SCgz/China	38.8	38.4	38.1	75.2	77.8	71.1	56.9	**98.3**	85.0
	TTSuV2-3/2022/SCgz/China	38.7	37.9	39.4	**92.4**	80.7	71.5	57.4	75.5	74.7
ORF2	TTSuV2-1/2022/SCgz/China	35.3	35.3	33.6	44.5	44.1	43.3	**99.2**	46.2	45.0
	TTSuV2-2/2022/SCgz/China	40.8	44.5	38.2	79.8	91.6	87.8	45.4	96.2	**97.1**
	TTSuV2-3/2022/SCgz/China	42.9	43.3	42.4	**97.1**	79.8	80.3	44.1	80.3	78.6

^1^ The highest nucleotide identities of different regions are indicated in bold typeface. Furthermore, the nucleotide similarity of the ORF1 and ORF2 genes in the three novel TTSuV strains and in nine representative strains of TTSuV 1, 2a, and 2b were compared. The results showed that ORF1 of TTSuV2-1/TTSuV2-2 shared the highest nucleotide similarity, 96.5%/98.3%, with TTSuV 2 strain 38E19/TTV2Bj1-2, while ORF1 of TTSuV2-3 shared the highest nucleotide similarity, 92.4%, with TTSuV 1 strain pork_298. ORF2 of TTSuV2-1/TTSuV2-2 shared the highest nucleotide similarity, 99.2%/97.1%, with TTSuV 2 strain 38E19/TTV2Bj1-2, while ORF1 of TTSuV2-3 shared the highest nucleotide similarity, 97.1%, with TTSuV 1 strain pork_298 (Table 1). Phylogenetic analysis indicated that the three TTSuV strains in Tibetan pigs belonged to TTSuV 2 genotype and could be divided into two distinct sub-genotypes: 2a and 2b. The TTSuV2-1/2022/SCgz/China and TTSuV2-2/2022/SCgz/China belong to the TTSuV 2b sub-genotype, and strain TTSuV2-3/2022/SCgz/China was classified into the TTSuV 2a sub-genotype (Figure 2).

#### 3.2.2. PCV-2

A total of 8,666,834 reads with sequences corresponding to porcine circovirus were detected in the pooled samples. Two complete genomes of porcine circoviruses were assembled and designated PCV2-1/2022/SCgz/China (1767 bp) and PCV2-2/2022/SCgz/China (1731 bp). These sequences were submitted to the GenBank database (accession no. OQ874786 and OQ656424). Similarity analyses revealed that the two porcine circovirus strains shared 95.1~99.1% identity with the PCV-2 reference strains, but only 40.1% to 57.2% identity with the PCV-3 and PCV-4 reference strains (Table 2). Furthermore, the nucleotide similarity was also compared for the Rep and Cap genes in the two novel PCV strains and in nine representative strains of PCV 2, 3 and 4. The results showed that both *Rep* and *Cap* genes of PCV2-1/PCV2-2 shared the highest nucleotide similarity, 98.6~99.0% and 99.2~99.4%, respectively, with PCV2d strains (Table 2).

**Figure 3:**
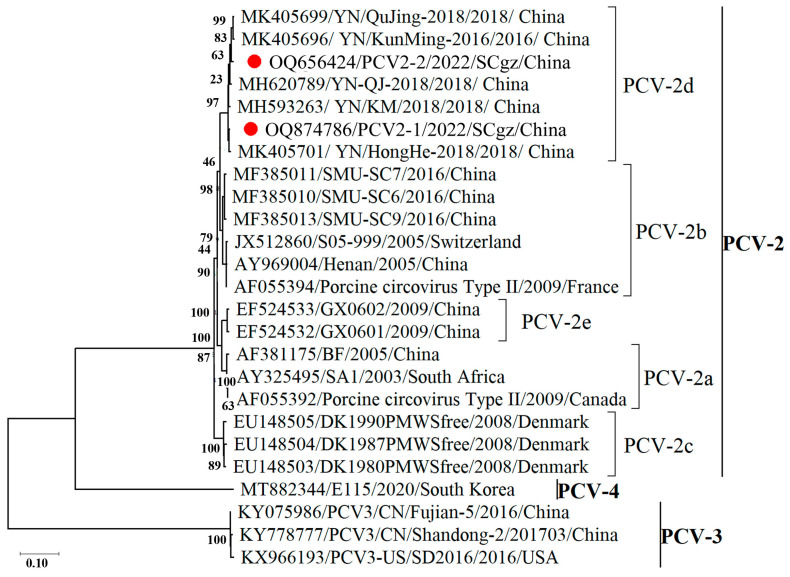
Phylogenetic tree based on the genomic sequence of two PCV strains identified in Tibetan pigs with 23 reference strains including PCV 2, 3, and 4. The three TTSuV strains in this study are labeled with “red circle”. Numbers along branches are bootstrap values. Scale bar indicates nucleotide substitute per site.

Phylogenetic analysis indicated that the PCV reference strains in this study were clustered into three genotypes: PCV-2, PCV-3, and PCV-4. The PCV-2 strains all showed evidence of evolutionary divergence and were further classified into five sub-genotypes (2a, 2b, 2c, 2d, and 2e). The two PCVs in Tibetan pigs belonged to the PCV-2 genotype and were further classified into the PCV-2d sub-genotype (Figure 3).

#### 3.2.3. Porprismacovirus

A total of 3,995,820 reads with sequences corresponding to Porprismacovirus were detected in the respiratory samples. One complete genome of Porprismacovirus was assembled and designated as PPMV-SCgz-2022. The sequences were submitted to the GenBank database (accession no. OQ656300), and the full-length genomic sequence was 2398 nucleotides in length.

Analysis using the MegAlign program of the DNAstar software (v7.1.0) revealed that the complete genome of PPMV-SCgz-2022 shared the highest nucleotide similarity (89.5%) with the human-associated porprismacovirus isolate 16,806 × 66-213 (GenBank accession no. MH111125), reported in Vietnam in 2013, but only 61.0–63.4%, 37.0–37.2%, 42.4–42.7%, and 35.7–42.2% identity with reference strains from domestic pigs, Chicken/Turkey, Rat, and Monkey/Chimpanzee, respectively. Interestingly, the Cap gene of PPMV-SCgz-2022 had the highest similarity (87.4%) with the human-origin strain 16,806 × 66_213, but the Rep gene had higher similarity (87.7–90.3%) to the swine-origin strains (Table 3).

**Figure 4:**
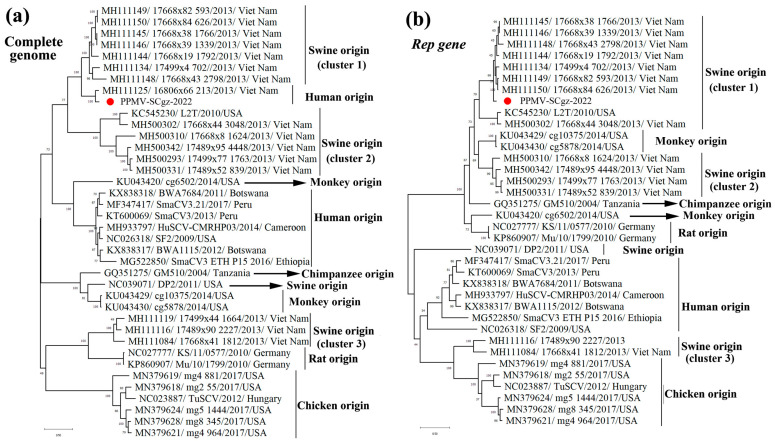
Phylogenetic trees based on complete genomic sequence (**a**) and Rep gene sequence (**b**) of the PPMV-SCgz-2022 strain identified in Tibetan pigs with reference strains isolated from different hosts. The PPMV-SCgz-2022 strain in this study is labeled with “red circle”. Numbers along branches are bootstrap values. Scale bar indicates nucleotide substitute per site.

Phylogenetic analysis of PPMV-SCgz-2022 was performed based on the complete genome and Rep gene sequences of reference PPMVs originated from different hosts. The results showed that the PPMV strains that originated from pigs had higher genetic diversity than those from other hosts and were divided into three different clusters (Clusters 1, 2, and 3). Phylogenetic trees showed that PPMV-SCgz-2022 was classified in the human-associated porprismacoviruses based on the full-length genomic sequence, but clustered into a swine-related branch of the phylogenetic tree according to the Rep genotyping (Figure 4). These findings suggested possible recombination between different hosts shaped the genome of PPMV-SCgz-2022.

### 3.3. Viral Detection in Lung Samples from Tibetan Pigs

Among the 18 detected viruses, 3 viruses were previously associated with PRDC: PCV-2, TTSuV, and PCMV. Furthermore, lungs from the 66 asymptomatic samples and 101 PRDC-positive pigs were subjected to PCR amplification and sequencing to verify the detection rates of PCV-2, TTSuV, and PCMV. The results showed detection of PCMV in 4.5% of the 66 asymptomatic samples, but the samples were negative for PCV-2 and TTSuV. From the 101 PRDC-positive cases (Table 4), the positive rates of PCV-2, TTSuV, and PCMV were 22.8% (23/101), 22.8% (23/101), and 3.0% (3/101), respectively. The rates of co-infection with PCV-2 + TTSuV, PCV-2 + PCMV, and TTSuV + PCMV were 13.9% (14/101), 3.0% (3/101), and 2.0% (2/101), respectively. PCV-2/TTSuV/PCMV co-infection was not detected in these samples. Among PCV2-positive samples, the rate of detection of PCV2 + TTSuV was 65.2% (15/23), but that of PCV2 + PCMV was only 4.3% (1/23).

## 4. Discussion

PRDC is a multifactorial syndrome that significantly affects the respiratory system of pigs, with important effects on the global swine industry [2]. Our metagenomics analysis detected mainly small linear and circular DNA viruses, with a total of 18 distinct viruses belonging to 15 viral taxonomic families present in the respiratory samples of Tibetan pigs with PRDC in this high-altitude area in China, which shows that the viral flora in these animals were complex and diverse. Among the 18 detected viruses, pathogens associated with PRDC in pigs, including PCV-2, TTSuV, and PCMV were detected [3,5,27,28]. However, few mammalian-associated viral sequences were detected in Tibetan pigs from asymptomatic pigs. This result may be related to the process of sample collection in the asymptomatic pigs. Compared with the PRDC group, all 66 samples in the control group were from nasal swabs from asymptomatic pigs. The nasal cavity of Tibetan pigs is relatively shorter than that of domestic pigs, making it difficult for swabs to penetrate deep into the nasal cavity for sufficient sample collection. In addition, due to the arid climate and low temperature in plateau areas, the nasal cavity of Tibetan pigs is quite dry, so it may be difficult to detect viruses using this collection method. Notably, PRRSV, as one of the primary pathogens of PRDC [3], was identified as negative in the 101 respiratory samples from Tibetan pigs with PRDC by RT-PCR. A previous study has demonstrated that Tibetan pigs are much less susceptible to PRRSV than domestic pigs [29], and, to our knowledge, there have been no clinical reports of PRRSV infection in Tibetan pigs. Interestingly, the low sequence read abundance of human gamma herpesvirus 4 (4.39%), fowl aviadenovirus (2.11%), human betaherpesvirus 7 (0.95%), and tylonycteris bat coronavirus (0.83%) indicates a possible relationship of disease in these animals with the unique farming model of Tibetan pigs in this region. The farming model may facilitate the cross-species transmission of viruses between different animals or the occurrence of zoonotic diseases, which can have important consequences for disease spread.

PCV2 was first identified in piglets in 1998 [30] and is associated with reproductive disorders, respiratory diseases, diarrhea, porcine dermatitis and nephropathy syndrome (PDNS), and postweaning multisystemic wasting syndrome (PMWS), causing considerable economic losses to the swine industry worldwide [31]. In this study, the PCV2 positivity rate in Tibetan pigs was 22.8% (23/101), significantly lower than the detection rate (72.22%) of respiratory samples from domestic pigs in low-altitude areas in Sichuan province [6]. Currently, eight sub-genotypes (PCV2a-PCV2h) have been identified in PCV2 strains with a genetic p-distance of 0.035 [32]. Since 2012, PCV2d has gradually replaced PCV 2a and PCV2b as the predominant sub-genotype in China [31]. Phylogenetic analysis indicated that the two PCV strains identified in Tibetan pigs belong to the PCV2d sub-genotype, which differs from the PCV2b sub-genotype we identified in Tibetan pigs in 2018 [10], showing similar prevalent trends in other geographic regions in domestic pigs in China.

TTSuV is a small circular single-stranded DNA virus with high population diversity [32]. Two TTSuV genotypes are currently prevalent in domestic pigs: Torque teno sus virus 1 (TTSuV1) and Torque teno sus virus 2 (TTSuV2) [7]. This is the first identification of TTSuV in Tibetan pigs. Phylogenetic analysis indicated that all TTSuV strains detected in Tibetan pigs belong to TTSuV2 and could be further divided into TTSuV2a and TTSuV2b sub-genotypes. Previous studies have shown that TTSuV can serve as an enhancing factor of co-infection with porcine circovirus and respiratory disease viruses, particularly PCV2 [7,33,34]. In this study, the TTSuV in Tibetan pigs showed the same positive rate as PCV2 (22.8%), and among PCV2-positive samples, the proportion of PCV2/TTSuV2 co-infection was as high as 65.2% (15/23), suggesting it is likely the main cause of PRDC in Tibetan pigs.

**Figure 5:**
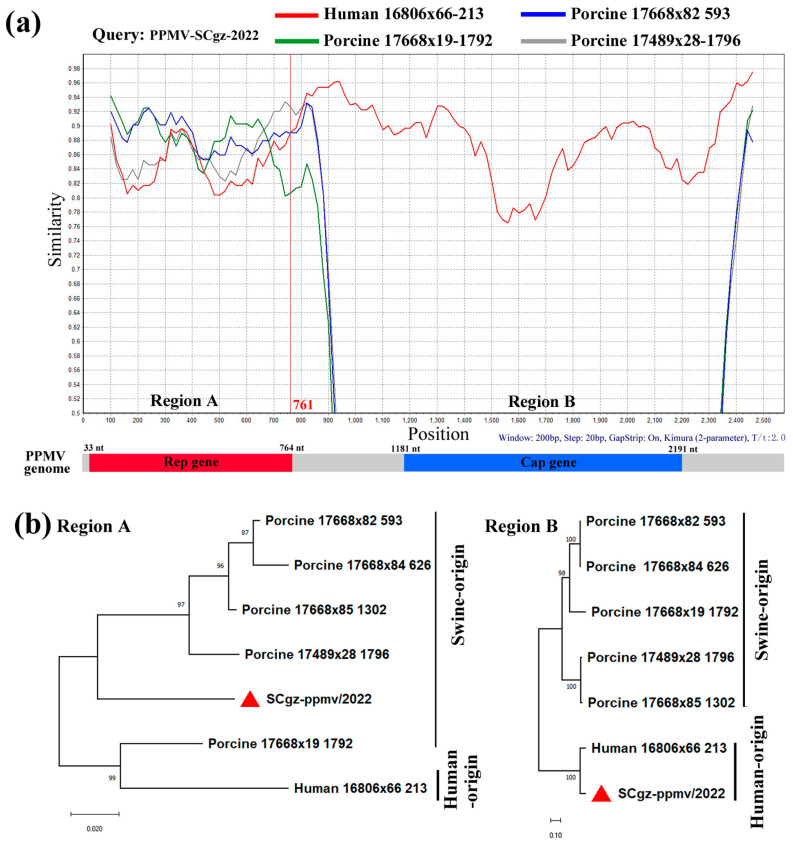
Recombination analysis of strain PPMV-SCgz-2022. (**a**) Genome scale similarity comparisons of PPMV-SCgz-2022 (query) with Human 16,806 × 66-213 (red), Porcine 17,668 × 82-593 (blue), Porcine 17,668 × 19-1792 (green), and Porcine 17,489 × 28-1796 (gray). One supposed recombination breakpoint within Rep gene and two recombination regions (Region A and B) were marked at the bottom with nucleotide sites. (**b**) Phylogenetic trees based on the two recombinant fragments within PPMV-SCgz-2022 were shown below the similarity plot. The PPMV-SCgz-2022 strain was labeled with “red triangle”.

Recently, the sequences of circular replication initiation protein-encoding single-stranded (CRESS) DNA viruses detected from eukaryotic hosts were found to have an abundant genetic diversity [35]. According to the latest taxonomy of viruses by the International Committee on Taxonomy of Viruses (ICTV), the *Smacoviridae* is a new family of animal-associated CRESS DNA viruses. There are at least 41 species within six new genera (*Bovismacovirus*, *Cosmacovirus*, *Dragsmacovirus*, *Drosmacovirus*, *Huchismacovirus*, and *Porprismacovirus*) in this family, and these species have been found in a wide range of animals [35,36]. The Porprismacovirus belong to the genus porprismacovirus of Smacoviridae family and have been identified in feces samples from various vertebrates, including human, porcine, chimpanzee, monkey, bovine, camel, chicken, rat, and sheep [37]. To identify possible recombination events in the genome of PPMV-SCgz-2022, we detected recombination using RDP4 (v4) and SIMPLOT (3.5.1) software. The analysis revealed that PPMV-SCgz-2022 sequences showed remarkably high degrees of certainty of a recombination event supported by RDP (3.443 × 10^−11^), BootScan (1.270 × 10^−19^), MaxChi (4.508 × 10^−16^), Chimaera (1.149 × 10^−4^), 3Seq (7.771 × 10^−15^), with all *p*-values < 0.01. From the similarity plot, one recombination breakpoint was identified within the PPMV-SCgz-2022 genome, located in the Rep gene (nt 761) (Figure 5a). This breakpoint separated the genome of PPMV-SCgz-2022 into two regions, where region A (nt 1-761) was closely related to swine-origin porprismacoviruses, and region B (nt 762-2575) was closely related to human-origin porprismacoviruses (Figure 5b). Collectively, the results indicated that PPMV-SCgz-2022 may have originated from recombination events between Human 16,806 × 66-213 strain and Porcine 17,668 × 82-593 strain. Of note, all reported porprismacovirus strains were identified from the fecal matter of various animals using metagenomic methods [36], suggesting that this virus likely is associated with animal diarrhea disease. However, in the present study, PPMV-SCgz-2022 was identified in lung samples from Tibetan pig with PRDC, indicating that the porprismacovirus may also be related to respiratory disease. To date, no porprismacovirus strains have been cultured, limiting further investigation of their pathogenicity.

## 5. Conclusions

In conclusion, this is the first report of the viral communities in PRDC-affected Tibetan pigs in the plateau region. The results revealed the complexity and diversity of the viral flora in these animals, and indicate that single infection or co-infection of PCV2 and TTSuV2 are the main causes of PRDC disease in Tibetan pigs. Sequence analysis based on the viral genomic sequences indicated that the PCV2 strains belonged to PCV2d, and TTSuVs were classified as TTSuV2a and TTSuV2b genotypes. Because Porprismacoviruses are newly discovered zoonotic viruses that can infect various animals and humans, surveillance of these viruses is of great significance for veterinary and public health.

## Figures and Tables

**Figure 2:**
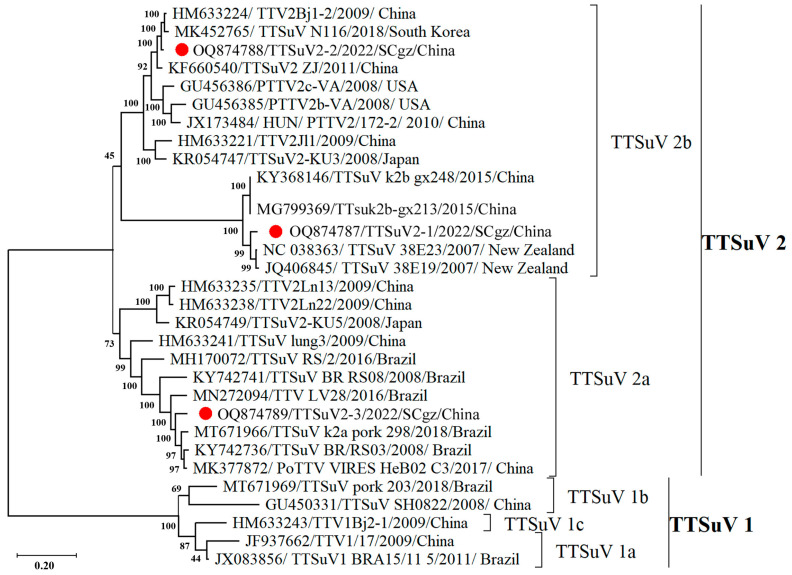
Phylogenetic tree based on the genomic sequence of three TTSuV strains identified in Tibetan pigs with 27 reference strains available in GenBank. The three TTSuV strains in this study are labeled with “red circle”. Numbers along branches are bootstrap values. Scale bar indicates nucleotide substitute per site.

**Table 2:** Nucleotide sequence identity values for different regions of two PCV strains identified in Tibetan pigs compared with nine PCV reference strains.

		SA1	Henan PCV2	DK1980PMWSfree	GX0602	QuJing	YN/QuJing-2018	PCV3-US/SD2016	PCV3/CN/Fujian-5/2016	E115
		PCV 2a	PCV 2b	PCV 2c	PCV 2e	PCV 2d		PCV 3		PCV 4
		Pairwise % Identity to TTSuV (nt)								
Complete genome	PCV2-1/2022/SCgz/China	95.9	97.0	95.3	95.6	**99.0** ^1^	98.8	40.2	40.1	56.8
	PCV2-2/2022/SCgz/China	95.8	96.7	95.1	95.4	99.0	**99.1**	40.3	40.1	57.2
Rep gene	PCV2-1/2022/SCgz/China	98.4	97.8	98.1	98.0	**98.6**	97.9	46.6	46.4	50.7
	PCV2-2/2022/SCgz/China	98.6	97.8	98.1	98.1	98.8	**99.0**	46.7	46.4	51.2
Cap gene	PCV2-1/2022/SCgz/China	90.9	94.9	89.8	90.2	99.3	**99.4**	40.2	40.2	53.0
	PCV2-2/2022/SCgz/China	90.4	94.4	89.3	89.8	99.0	**99.2**	40.3	40.3	53.1

^1^ The highest nucleotide identities of different regions are indicated in bold typeface.

**Table 3:** Nucleotide and amino acid homology between SCgz-2022 strain and 14 reference strains isolated from different hosts.

Host	Strains	Country/Year	Complete Genome (%)	*Rep* Gene (%)	*Cap* Gene (%)
Swine	17,668 × 82_593	Vietnam/2013	62.2	89.6	45.4
	17,668 × 19_1792	Vietnam/2013	61.0	87.7	43.5
	17,489 × 28_1796	Vietnam/2013	63.4	**90.3** ^1^	37.3
	17,668 × 85_1302	Vietnam/2013	62.9	89.5	37.4
Human	16,806 × 66_213	Vietnam/2013	**89.5**	86.3	**87.4**
	Smacv3	Peru/2013	39.0	72.6	47.4
	SF2	USA/2009	38.6	73.1	48.6
Chicken/Turkey	mg2_55	USA/2017	37.2	75.8	46.1
	mg8_345	USA/2017	37.2	55.1	45.7
	TuSCV	Hungary/2012	37.0	72.7	44.5
Rat	Mu/10/1799	Germany/2010	42.4	52.0	50.2
	KS/11/0577	Germany/2010	42.7	52.4	50.2
Monkey/Chimpanzee	cg5878	USA/2014	35.7	60.8	38.7
	GM510	Tanzania/2004	42.2	52.6	41.1

^1^ The highest nucleotide identities of different regions are indicated in bold typeface.

**Table 4:** Viruses detected by PCR in 101 respiratory samples from Tibetan pigs in four regions.

Region	Pigs with PRDC	No. Positive of Pigs with PRDC					
		PCV-2	TTSuV	PCMV	PCV-2 + TTSuV	PCV-2 + PCMV	TTSuV + PCMV
Luding	31	8	7	1	4	1	1
Kangding	47	9	13	2	7	2	1
Daocheng	15	3	1	0	1	0	0
Xiangcheng	8	3	2	0	2	0	0
rate (positive/total)	101	22.8% (23/101)	22.8% (23/101)	3.0% (3/101)	13.9% (14/101)	3.0% (3/101)	2.0% (2/101)

## Data Availability

The genome sequences of TTSuV2-1/2022/SCgz/China, TTSuV2-2/2022/SCgz/China, TTSuV2-3/2022/SCgz/China, PCV2-1/2022/SCgz/China, PCV2-2/2022/SCgz/China, and PPMV-SCgz-2022 have been deposited in the GenBank database (19-MAR-2023) under the accession number of OQ874787, OQ874788, OQ874789, OQ874786, OQ656424, and OQ656300, respectively.

## Supplementary Materials

The following supporting information can be downloaded at: https://www.mdpi.com/article/10.3390/pathogens13050404/s1, Table S1: Specific primers used to detect the viruses of PCV2, TTSuV1, TTSuV2, and PCMV in this study.
