# Supplementary material for: Metagenomics to Identify Viral Communities Associated with Porcine Respiratory Disease Complex in Tibetan Pigs in the Tibetan Plateau, China
Source: Pathogens. 2024 May 13;13(5):404. doi: 10.3390/pathogens13050404 (PMC11124006; doi:10.3390/pathogens13050404)
Supplement: Supplementary file 1 [file pathogens-13-00404-s001.zip › pathogens-2963852-supplementary.pdf]

Supplementary Material

1 Supplementary Figures and Tables

1.1 Supplementary Tables

Table S1 Specific primers used to detect the viruses of PCV2, TTSuV1, TTSuV2, and PCMV in this study

Virus	Primer name[a]	Primers sequence (5´-3´)	Length (bp)
PCV2	PCV2-F	GAAATCAACTCTGGCTGAGAC	547
	PCV2-R	CAAGTGGTGGGATGGTTAC	
TTSuV1	TTSuV1-F	TACACTTCCGGGTTCAGGAGGCT	318
	TTSuV1-R	ACTCAGCCATTCGGAACCTCAC	
TTSuV2	TTSuV2-F	AGTTACACATAACCACCAAACC	264
	TTSuV2-R	ATTACCGCCTGCCCGATAGGC	
PCMV	PCMV-F	CATCAGAGCCACGAAAT	579
	PCMV-R	GGAGAAGCATAGTAACCC	

[a] F and R represent forward and reverse primers, respectively.
